# Effects of Different Roasting Methods on the Quality of Roasted Large Yellow Croaker (*Larimichthys crocea*)

**DOI:** 10.3390/foods13172772

**Published:** 2024-08-30

**Authors:** Chenjing Yin, Chao Zhang, Yangli Xu, Laijin Su

**Affiliations:** 1 College of Life and Environmental Science, Wenzhou University, Wenzhou 325035, China; 18850637059@163.com (C.Y.);; 2 Zhejiang Provincial Key Laboratory for Water Environment and Marine Biological Resources Protection, Wenzhou University, Wenzhou 325035, China; 3 Wenzhou Academy of Agricultural Science, Wenzhou Characteristic Food Resources Engineering and Technology Research Center, Wenzhou 325006, China

**Keywords:** large yellow croaker roasted fish, roasting method, texture properties, moisture-distribution, volatile flavor compounds

## Abstract

This study investigated the effects of different roasting methods (45% light wave and 55% microwave roasting, 70% light wave and 30% microwave roasting, 100% light wave roasting, far-infrared roasting, and oven roasting) on the quality of roasted large yellow croaker. The quality was evaluated using sensory evaluation, texture characteristics, color differences, moisture content, and volatile flavor substances. In this context, different roasting methods can affect the color, taste, and flavor of large yellow croaker fish, significantly improving the overall acceptance of roasted fish. The results showed that after 45% light wave and 55% microwave roasting, the elasticity of fish meat was maintained, the hardness of fish meat was reduced, the moisture content and distribution were changed, and the taste was the best. Far-infrared roasting and 45% light wave and 55% microwave roasting had a significant effect on the color of large yellow croaker samples and improved the sensory evaluation score. Forty-six volatile compounds were detected using gas chromatography–mass spectrometry. After roasting, the oxidation and Maillard reactions of lipids and proteins were increased, with the 45% light wave and 55% microwave roasting giving the highest variety of volatile flavor substance products.

## 1. Introduction

The large yellow croaker (*Larimichthys crocea*) is a shallow sea fish belonging to the genus *Larimichthys* in the order Perciformes. It is also known as yellow croaker, cucumber fish, and golden dragon. The large yellow croaker is distributed in the coastal waters of the East China Sea and the South Yellow Sea, South Korea, Japan, Vietnam, and in the Northwest Pacific Ocean. The large yellow croaker fishing industry once dominated China’s four major fisheries (the others are small yellow croaker, hairtail, and cuttlefish) [1]. Large yellow croaker meat contains amino acids, protein, fat, and niacin, with the effect of tonifying the air and blood, fitness, and beauty [2]. It is favored by consumers in China and Southeast Asia [3]. According to the statistics, the total output of large yellow croaker in China is increasing year by year. In 2022, the total aquaculture output will be 257,700 tons, ranking first in China’s marine fish aquaculture industry [4]. This type of fish is usually processed into various products by freezing, pickling, and drying [5]. The products include pickled, smoked, and distiller-processed large yellow croaker products and snack food, but the development of roasted products is still limited [6].

Roasted fish is an instant, hot food that is convenient and fast to consume. It combines the three cooking techniques of pickling, roasting, and stewing [7]. Grilled fish is rich in nutrients, has a desirable flavor, and has a consumption history that can be traced back more than 1000 years. However, according to one survey, China’s grilled fish market is expected to reach approximately 140 billion yuan by 2023 [8]. Traditional cooking methods for fish often involve frying at high temperatures. After frying, eicosapentaenoic acid and DNA in the grilled fish are damaged, and the protein and fat in the fish undergo oxidation, hydrolysis, cracking, cyclization, and polymerization. This accelerates the loss of water, which affects the sensory, textural, and flavor of cooked fish products and has an important impact on the formation of harmful substances, such as trans fatty acids and polycyclic aromatic hydrocarbons [9]. To avoid the production of excessive polycyclic aromatic hydrocarbons by traditional high-temperature frying methods and reduce the water loss rate [10], new processing methods, such as far-infrared roasting, microwave roasting, and light wave roasting, can be considered. Far-infrared roasting adopts the principle of infrared radiation wavelength drying and heating, which effectively prevents fish from overheating and oxidizing. It reduces the decomposition of nutrients and the processing loss rate and provides uniform and rapid heating [11]. Microwave roasting uses the principle of the thermal heating effect. Microwave energy can be transferred to the interior of the food, and the liquid water inside the food is vaporized into water vapor resulting in the overflow of water molecules, which can adjust the stability of the temperature difference between the inside and outside of the fish, thereby improving the color and texture characteristics of the food. Light wave combination is a new type of baking technology [12]. Light wave baking is used to bake the surface of the food, while microwave acts on the internal heating of the food. It uses the combination of infrared light wave and microwave light wave to alternately act on the internal parts and surface of the food, and has the characteristics of high efficiency and fast and uniform heating.

In this study, five different roasting methods, namely, 45% light wave and 55% microwave roasting (rated power of 767.5 W), 70% light wave and 30% microwave roasting (rated power of 770 W), 100% light wave roasting (rated power of 850 W), far-infrared roasting (upper fire 180 °C, lower fire 200 °C, rated power of 2000 W), oven roasting (160 °C, rated power of 2000 W) were tested for their effects on the sensory and texture characteristics, color, water content and migration, and flavor characteristics of large yellow croaker fish meat. The purpose of this study was to determine the effects of different roasting methods on the quality and flavor of large yellow croaker fish and to provide a basis for the optimization of roasting methods and flavors.

## 2. Materials and Methods

### 2.1. Materials and Instruments

The following materials were used: fresh large yellow croaker (Wenzhou City, Zhejiang Province, China) and edible salt (Shandong Daiyue Salt Co., Ltd., Qingdao City, Shandong Province, China).

The following instruments were used: far-infrared oven (CRDF32WBL; Guangdong Weishida Electrical Technology Co., Ltd., Guangzhou City, Guangdong Province, China), microwave oven (G70F20CN1L-DG [B0]; Guangdong Galanz Group Co., Ltd., Guangzhou City, Guangdong Province, China), electric hot-blast constant-temperature drying oven (101-1BS; Shaoxing Supo Instrument Co., Ltd., Wenzhou City, Zhejiang Province, China), meat grinder (Zhejiang Supor Co., Ltd., Wenzhou City, Zhenjiang Province, China), texture analyzer (TMS-PRO; Food Technology Corporation, Boston, MA, USA); colorimeter (NH300; Shenzhen Ruihan Technology Co., Ltd., Shenzhen, China), nuclear magnetic resonance analyzer (MacroMR20-060H-I; Suzhou Newmai Analysis Co., Ltd., Suzhou, China), and gas chromatography–ion mobility spectrometer (GC-IMS; FlavourSpec^®^; Hainan Instrument Co., Ltd., Shanghai, China).

### 2.2. Sample Preparation

**Figure 1:**
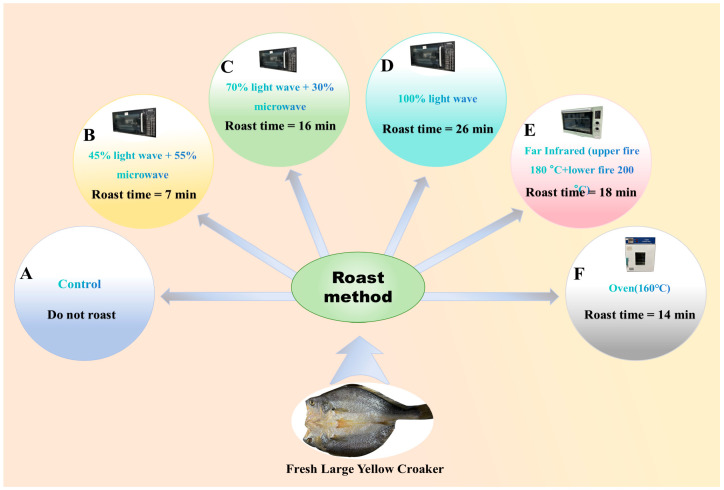
The best parameters of different large yellow croaker fish roasting methods. A–F represent different roasting methods. A—control, B—45% light wave and 55% microwave roasting (45% and 55% represent the values of light wave and microwave fire power), C—70% light wave and 30% microwave roasting (70% and 30% represent the values of light wave and microwave fire power), D—100% light wave roasting, E—far-infrared roasting, and F—oven roasting.

Fresh large yellow croakers of suitable size were selected as the raw materials. After slaughter, the gills, scales, and internal organs were removed. The fish were cut open along the back line and rinsed three to four times with clean water. After cleaning, the fish were placed in a pickling solution with 1.5% salt water. After pickling at 4 °C for 6 h, the fish were removed and rinsed with water, and the water was drained. All procedures were performed in accordance with the instructions of the World Organization for Animal Health (OIE) guidelines. It also complies with the ethical exemption requirements of the *Measures for Ethics Review of Human Life Science and Medical Research issued* by China. Based on the comprehensive sensory evaluation scores of the five roasting methods in the preliminary experiment, the parameters with the highest scores for each roasting method were selected and marked as groups A, B, C, D, E, and F. The specific optimal roasting parameters are presented in Figure 1. All samples of roasted large yellow croaker (20 g) up to 5 min after heat treatment were portioned to food-grade vacuum bags, then vacuum packaged, and encoded. Thereafter, they were placed in thermally insulated Styrofoam boxes to maintain the temperature (37 ± 1 °C) and delivered for evaluation.

### 2.3. Determination of the Color Difference Value

The color difference was determined according to the method of Abdel-Naeem et al. [13]. The processed large yellow croaker grilled fish samples were placed in an uncovered transparent cylinder mold with a diameter of 4 cm and a height of 2 cm. The color difference meter was placed vertically with the surface of the subject, and the values of lightness (L*), redness–greenness (a*), and yellowness–blueness (b*) were measured three times. The average value was then recorded.

### 2.4. Determination of Texture Value

The texture difference was determined according to the method of Haghighi et al. [14]. Muscle texture was measured using a texture instrument. The hardness, viscosity, elasticity, and chewiness of the meat were analyzed using two compression texture profile analysis modes. The texture analyzer parameters were as follows: dwell time, 5 s; pressure, 100 N; distance, 30 mm; deformation, 20%; speed, 60 mm/s; initial pressure, 1 N; and interval time, 5 s. 

### 2.5. Determination of Water Distribution 

Water distribution and migration were determined according to the method described by Qian et al. [15], with slight modifications. The grilled fish muscle was cut into 2.0 cm × 2.0 cm × 1.0 cm pieces and put into a nuclear magnetic tube.

The CPMG sequence was as follows: SW = 100 KHz, SF = 21 MHz, O1 = 34,171 Hz, P1 = 5.8 us, P2 = 10.24 us, RFD = 0.08 ms, RG 1 = 20.0 db, DRG 1 = 3, PRG = 1, TE = 0.1 ms, NECH = 5000, TW = 30,000 ms, and NS = 16.

The inversion parameters were as follows: SRIT model, smoothing factor = 0, T_min_ = 0.01, T_max_ = 10,000, and 30,000 iterations.

The SE sequence was as follows: FOV = 50 * 50 mm, TR = 1000 ms, TE = 7.64 ms, read size = 256, phase size = 128, slice count = 3, slice thickness = 2 mm, slice gap = 0.5 mm, averages = 6, and echo position = 30%. After iterative inversion of the transverse relaxation time T2 map, each sample determination was performed in parallel 3 times.

### 2.6. Determination of Volatile Flavor Compounds

Volatile flavor substances were determined according to the method described by Sheng et al. [16], with slight modifications. Volatile compounds with flavors were separated and identified by gas chromatography–ion mobility spectrometry (GC-IMS). After headspace injection, the volatile organic components in the roasted large yellow croaker fish were quickly detected and measured. The operational settings of the GC-IMS instrument were as follows: headspace incubation temperature, 60 °C; incubation time, 20 min; rotation speed, 250 rpm; injection needle temperature, 65 °C. The injection speed was set to 60 mL/min. The amount of sample injected was 5 g, the injection volume was 0.8 mL, and the non-shunt mode was used. The gas chromatography conditions were set as follows: column temperature, 60 °C; running time, 25 min; carrier gas, high-purity N_2_ (purity ≥ 99.99%); and initial flow rate, 2.0 mL/min for 2 min, increasing to 150 mL/min after 25 min. The IMS conditions were as follows: polar column, MXT-WAX (15 m, 0.53 mm internal diameter, 1 μm df); drift gas (N_2_, purity ≥ 99.99%); flow rate, 150 mL/min; and IMS temperature, 60 °C.

### 2.7. Sensory Evaluation of Roasted Large Yellow Croaker Prepared Using Different Roasting Methods

A sensory evaluation of the roasted fish was carried out to evaluate the shape, smell, color, taste, and acceptance. Ten experienced sensory personnel were selected for the evaluation. Five males and five females aged between 21 and 30 years performed the evaluations. They were healthy and disease-free and had a normal sense of smell, vision, and taste. The sensory evaluation criteria for the fried fish are shown in Table 1.

### 2.8. Statistical Analysis

The experiment was repeated three times, and the data are presented as the averages of the three parallel experiments. The results are expressed as the mean ± standard deviation. The SPSS Statistics software (version 26.0; IBM, Armonk, NY, USA) was used for analysis of variance and the determination of the levels of significance (*p* < 0.05). VOCal was used to analyze and plot the data for the key flavor substances. T2 relaxation time spectra were obtained using the Origin 2024 software. Partial least squares discriminant analysis was performed using the SIMCA14.1 software.

## 3. Results and Discussion

### 3.1. Effects of Different Roasting Methods on the Color of Large Yellow Croaker

Color is an important criterion for the sensory evaluation of fish and is also an important determinant of consumers’ desire to buy [17]. L* represents the brightness of the color, a* represents the red–green value, and b* represents the yellow–blue value [18]. 

The experimental results of the color-difference measurements are shown in Table 2. Kong et al., reported a rapid whitening followed by a browning phase during thermal processing of pink salmon at 100 to 131.1 °C [19]. The browning phase usually appears as processing temperature and time increase [20]. In this study, as the temperature and roasting time increased, protein denaturation, lipid oxidation, and the Maillard reaction occurred in the fish [21]. The fish in group A were grey-white, with the highest L* value and the lowest a* and b* values. Compared to group A, the L* values of the five types of large yellow croaker meat roasted in different ways showed a downward trend, and the a* value showed an upward trend. This is because, with the increase in temperature or the extension of roasting time, the water evaporation of fish meat was accelerated, resulting in water loss from the surface, and the reaction and refraction of light were weakened, resulting in a decrease in the L* value [22]. With protein denaturation and lipid oxidation, the surface color of fish meat deepens to reddish-brown or golden yellow, resulting in an increase in the a* value. The L* values of large yellow croaker meat roasted using the three types of light waves or light wave–microwave combination were significantly lower than those of large yellow croaker meat roasted in the oven and using the far-infrared method (*p* < 0.05). It is speculated that the reason is that there is intense movement of the water molecules inside the fish as they are excited by microwaves, while the light waves act on the surface of the fish. The constant roasting time was long, and the temperature was high, which led to the greatest water loss on the surface of the fish [23]. As shown in Table 2, the L* value of the fish roasted under the five methods was most reduced by the 70% light wave and 30% microwave roasting method, which showed a value of 28.34. The a* value of large yellow croaker meat roasted with 100% light wave increased the most, with a value of 5.90, and was significantly different to the a* value obtained using the other roasting methods (*p* < 0.05). The b* value increased the most in the far-infrared-roasted meat, which showed a value of 9.51. This was because the Maillard reaction occurred during roasting. Far-infrared radiation had a certain penetration force and acted directly on the interior of the large yellow croaker meat [24,25]. When the temperature was the highest, the heat was uniform, and more brown substances were produced [26,27]. Therefore, the surface of the far-infrared-roasted fish was golden yellow and the b* value was the largest.

### 3.2. Effects of Different Roasting Methods on the Texture Characteristics of Roasted Large Yellow Croaker Fish 

In this study, the elasticity, hardness, chewiness, and adhesiveness of roasted large yellow croaker were analyzed. Texture is a key quality used by the processed food industry to evaluate product quality and consumer acceptability, while hardness is an important factor in measuring differences in fish taste [28]. The textural characteristics of the large yellow croaker meat obtained using the five roasting methods are listed in Table 3. The elasticity, hardness, chewiness, and adhesiveness of group A (raw fish) are the highest. There were no significant differences in the effects of the five roasting methods on the elasticity of the meat of the large yellow croaker (*p* > 0.05). Compared with control group A (raw fish), hardness and chewiness of the roasted large yellow croaker meat showed a downward trend, which was positively correlated with chewiness. The reason may be that after roasting, the protein is denatured, resulting in the destruction of the three-dimensional network structure, the loosening of the muscle fibers [29], the decrease in the cell binding capacity, and the softening of the fish texture. The hardness of fish in groups B, C, D, E, and F accounted for 36.00%, 39.00%, 49.84%, 36.80%, and 41.64% of the raw fish, respectively. The hardness of fish roasted by method B (45% light wave and 55% microwave) decreased the most. The reason may be that microwave stimulates specific molecules to penetrate the sample, resulting in cell rupture, the excitation of reactive oxygen species [30], and the stimulation of increased protein oxidation in meat products [31]. This showed that the large yellow croaker roasted with 45% light wave and 55% microwave had great elasticity, soft meat, was more chewy, and tasted good.

### 3.3. Effects of Different Roasting Methods on Water Migration in Large Yellow Croaker

**Figure 2:**
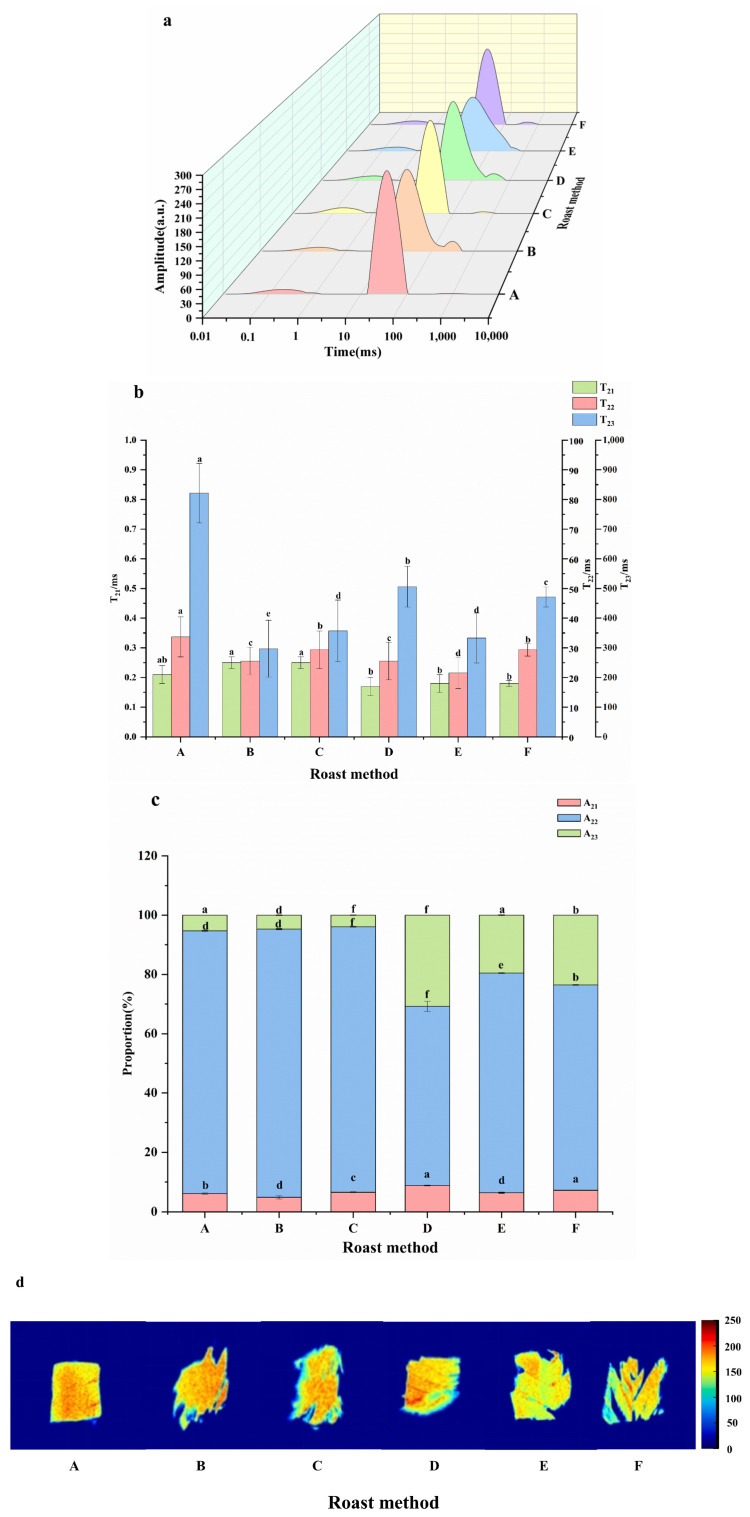
The moisture content and moisture distribution of large yellow croaker prepared with different roasting methods. The plots in (**a**–**d**) represent the low-field nuclear magnetic resonance (LF-NMR) T_2_ relaxation time spectrum, the T distribution relaxation time distribution, the corresponding peak area ratio, and the nuclear magnetic resonance imaging pseudo-color map of different roasting methods of large yellow croaker fish, respectively. A–F represent different roasting methods. A—control, B—45% light wave and 55% microwave roasting, C—70% light wave and 30% microwave roasting, D—100% light wave roasting, E—far-infrared roasting, and F—oven roasting. Different lowercase letters (a–f) in the same parameter indicate significant differences between different roasting methods (*p* < 0.05). “a”, “b”, “c”, “d”, “e”, “f” represents a significant difference between the groups. “a” and “ab” contain the same letter “a”, indicating no significant difference between the two groups. “b” in “ab” indicates no significant difference between this group and other groups containing the letter “b”.

Moisture is an important factor that affects the texture and flavor of food. A reduction in immobilized water affects the chewiness and hardness of large yellow croaker meat and causes a deterioration in its sensory quality [32,33]. In meat science research, T_2_ is commonly used to characterize relaxation time. The change in T_2_ can reflect the fluidity of water molecules, which is related to the degree of freedom and binding force of hydrogen protons in water. A larger T_2_ value indicates a higher degree of freedom of water, and a smaller T_2_ value indicates that the water is combined closer to the substrate [34,35]. The T_2_ inversion map of large yellow croaker meat subjected to different roasting methods is shown in Figure 2a, and three peaks can be observed: T_21_, T_22_, and T_23_. The range of T_21_ is 0.01–10 ms, which is considered to be closely attached to the polar groups on the surface of the fish protein molecules and it combines with biological macromolecules, such as proteins and lipids, to form a hydration layer, that is, bound water. T_22_ ranges from 10 to 100 ms and is considered to be the fixed water trapped in the myofibrillar structure, that is, the immobile water. T_23_ ranges from 100 to 1000 ms and is considered to be the free water in the myofibrillar structure [36]. Therefore, the total area of the low-field nuclear magnetic resonance (LF-NMR) inversion spectrum of large yellow croaker meat after different roasting methods tended to decrease, and the peak of the spectrum moved to the right, indicating that different roasting methods changed the water state of the meat, and most of the water in the large fish was fixed water [37,38].

The immobilized water and free water of the roasted fish changed significantly under different roasting methods (*p* < 0.05). As shown in Figure 2b, compared with control group A (raw fish meat), the T_21_ values of the roasted fish were not significantly reduced (*p* < 0.05). As shown in Figure 2c, the bound water content is very low, so it is considered to have little effect on the water holding capacity [39]. The T_22_ relaxation time of large yellow croaker meat was significantly shortened after roasting. Therefore, the degree of freedom of water in the fish was reduced, and the binding force was increased, indicating that the stability of the fixed water and free water was destroyed during the roasting process, which led to the migration and loss of water [40,41]. This was in agreement with the evaluation of marinated salmon flesh by Feng et al. using a combination microwave oven at 200 °C [42]. As shown in Table 4, the T_21_ value of light–microwave combined roasting in groups B and C increased significantly, indicating that during the roasting process, some of the immobile water was converted into bound water, which made the meat softer. The T_21_ value of group F oven roasting decreased, indicating that the bound water content of the large yellow croaker decreased during the roasting process. The reason may be that the cells of fish meat were destroyed during high-temperature heating, and the bound water flowed out. The immobilized water and free water volatilized rapidly through hot air roasting, which increased the hardness of the fish meat [43]. This shows that microwave and light wave roasting directly act on the small molecules inside the large yellow croaker fish meat and are evenly heated, which can make the fish meat fiber shrink evenly during the heating process, resulting in a certain elasticity of the fish meat, significantly improving the fluidity of the large yellow croaker’s immobilized water, which is conducive to the diffusion of internal water. Similar phenomena were discovered when cooking ridgetail white prawn (*Exopalaemon carinicauda*) [44].

To fully understand the dynamic changes and migration of the different aqueous phases, the values of A_21_, A_22_, and A_23_ corresponding to the quantitative data of the T relaxation times T_21_, T_22_, and T_23_, respectively, were calculated. As shown in Figure 2c and Table 5, the A_22_ values were the main water component, and the A_22_ values of the five roasting methods showed a downward trend, with significant differences (*p* < 0.05). Compared with control group A, the largest decrease in A_22_ value was seen for the 70% light wave- and 30% microwave-baked large yellow croaker meat, which decreased by 37.66%. The 45% light wave and 55% microwave roasting decreased the A_22_ value by 39.84%. The A_23_ values of the two roasted large yellow croaker fish decreased by 53.96% and 34.52%, respectively. This shows that the water molecules in the large yellow croaker meat after roasting in these two ways are lost. The reason may be because, during the 70% light wave and 30% microwave roasting process, the polar molecules inside the large yellow croaker meat absorbed the microwave energy and converted it into heat energy, which caused the fish meat to heat rapidly, and the whole sample was in a heated state. Owing to the water pressure difference between the fish tissue and the surrounding air, the water is evenly lost [45]. However, the A_22_ values of the other three roasting methods showed an opposite trend. This shows that far-infrared roasting, oven roasting, and 100% light wave roasting promote the conversion of fixed water to free water to a certain extent. The main reason is that the high temperature heating of the three roasting methods makes the protein denatured, resulting in the contraction of myofibrillar protein and myosin, causing the change in the water storage space structure, which in turn leads to the release of the water originally stored in myofibrils, which is converted into free water loss and reduces the rate of water migration. The texture of the fish meat baked using microwaves and light waves was clear, due to the effect of microwave heat on the interior of the material, which promotes the migration of water to the surface [46]. The torn surface of the fish baked in the oven was uneven and partially broken. Because blast-drying acts on the outside of the material, the water on the surface of the material can evaporate with time, so that the transfer of water and heat is more uniform, and the structure of the muscle fiber bundle is greatly improved. Therefore, the hardness of the fish baked using microwaves and light waves was low.

Magnetic resonance imaging (MRI) can be used to visualize the water distribution and internal structure of food samples and visually display changes induced by various food-processing methods [47]. Higher signal intensities at different points on the cross-section of the sample indicate greater proton enrichment, and these pixels are combined and pseudo-colored to obtain the magnetic resonance image [48]. Red represents a high proton density and blue represents a low proton density in 2-weighted images in the corresponding curve related to T strength. In Figure 2d, the changes in water distribution and internal structural information of raw fish and the five different roasted fish samples are displayed in the form of pseudo-colored images. The MRI results of all fish samples are red and blue. The signal of the fish meat roasted with microwaves and light waves was the highest among the five different roasting methods. Compared with the other three roasting methods, it takes longer to remove water from the fish meat, which may be related to its high bound-water content.

### 3.4. Effects of Different Roasting Methods on the Flavor Substances of Large Yellow Croaker

#### 3.4.1. GC-IMS Qualitative Analysis of Fish Samples Prepared Using Different Grilling Methods

**Figure 3:**
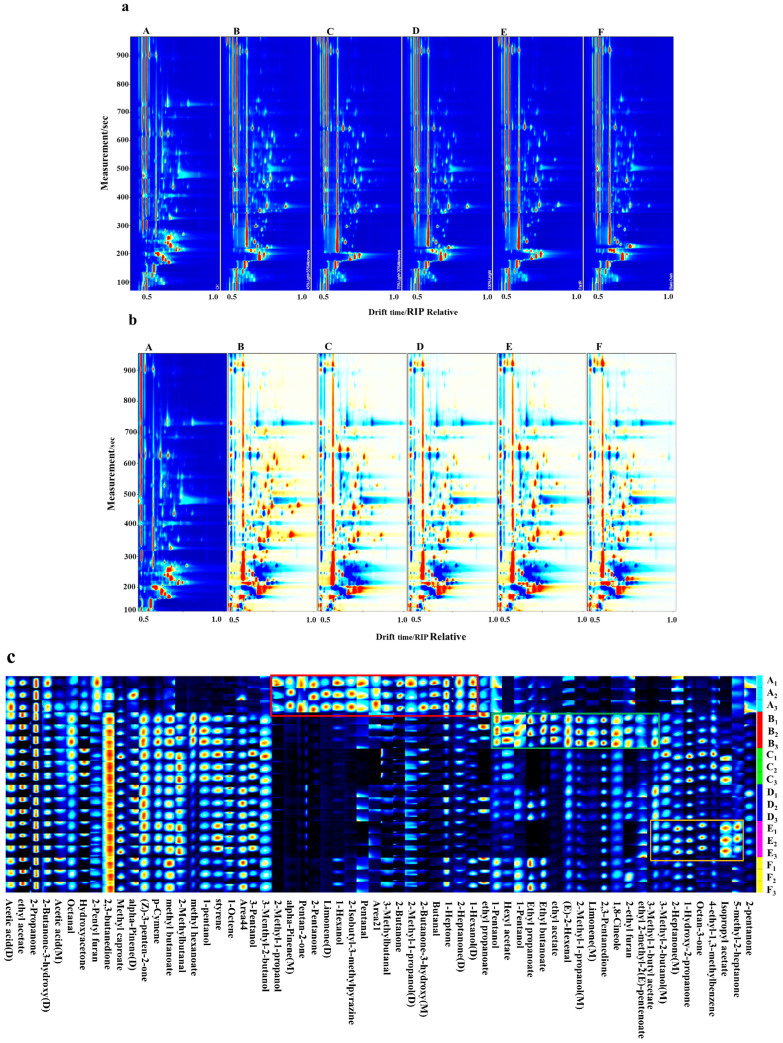
Gas chromatography–ion mobility spectrometer was used to analyze the changes in flavor characteristics of large yellow croaker roasted samples with different roasting methods. (**a**–**c**) represent two-dimensional topographic maps of large yellow croaker prepared using different roasting methods; (**a**) vertical view; (**b**) difference map; and (**c**) corridor characteristic fingerprint. A–F represents different roasting methods. A—control, B—45% light wave and 55% microwave roasting, C—70% light wave and 30% microwave roasting, D—100% light wave roasting, E—far-infrared roasting, and F—oven roasting.

Volatile organic compounds in the roasted fish were identified using GC-IMS. Figure 3a,b show raw fish sample A as a reference. The difference spectrum was obtained by comparing the samples obtained using the five roasting methods. The x-axis represents the drift time of the ions, and the y-axis represents the gas chromatography retention time. The concentration below the reference is shown in blue, the concentration equal to the reference is shown in white, and the concentration higher than the reference is shown in red [49,50]. The comparison diagram shows that there were significant differences in the compounds among the five roasting methods.

To further analyze the changes in volatile substances in large yellow croaker roasted using different roasting methods, all peaks were used to draw fingerprints. Each row in Figure 3c represents the signal peaks of the volatile compounds in the fish prepared using different roasting methods, and each column represents the signal peaks of the same volatile compounds in the fish roasted using different roasting methods. Each column compares the volatile compounds in different samples. The color intensity represents the content of volatile compounds, with a brighter color indicating a higher content [51,52].

#### 3.4.2. Volatile Compounds in Fish Samples Prepared Using Different Roasting Methods

GC-IMS was used to qualitatively analyze the volatile organic compounds formed during the roasting of large yellow croaker. As shown in Table 6, 46 volatile flavor components were identified in the five roasted fish samples and in the raw fish, with 25 substances detected in the raw fish. As shown in Figure 3c, 29, 22, 21, 20, and 20 flavor substances were detected in the 45% light wave and 55% microwave group (B), 70% light wave- and 30% microwave-baked group (C), 100% light wave-baked group (D), the far-infrared-baked group (E), and the oven-baked group (F), respectively. Alcohols, ketones, esters, aldehydes, and olefins were among the various flavor substances detected, including 12 ketones, 10 esters, eight alcohols, six aldehydes, five olefins, and five other compounds.

In Figure 3c, the red rectangular area indicates higher concentrations of various compounds in the raw fish meat of control group A, including valeraldehyde, hexanol, 2-butanone, 2-pentanone, 2-heptanone, 3-hydroxy-butanone, 2-methyl-1-propanol, 2-isobutyl-3-methylpyrazine, and limonene. There were significant changes in the following 15 flavor compounds after roasting: 2-butanone, 2-pentanone, 2-heptanone, 3-hydroxy-butanone, 1-hexanol, 2-methyl-1-propanol, propyl acetate, butyraldehyde, valeraldehyde, limonene, and pinene. After five roasting methods, 32 new flavor compounds were produced in the large yellow croaker, including 3-methyl-2-butanol, 3-pentanol, 1-pentanol, 2-methylbutanal, 1-octene, styrene, and methyl butyrate. The large yellow croaker meat baked using 45% light wave and 55% microwave in group B had the most types of flavor compounds, with ethyl acetate, ethyl butyrate, and hexyl acetate only existing in the fish baked using 45% light wave and 55% microwave.

The differences in the flavors of large yellow croaker meat prepared using different roasting methods were due to the differences in the roasting methods, temperature, and time, and the reactions in the fish meat were also different. Lipid oxidation, amino acid catabolism, esterification, and the Maillard reaction produced unique flavors and released a series of volatile substances.

#### 3.4.3. Aldehydes in Large Yellow Croaker Prepared Using Different Roasting Methods

Six aldehydes were detected in the meat of large yellow croakers. Valeraldehyde, isovaleraldehyde, and butyraldehyde were present only in the raw meat. Octanal, 2-hexenal, and 2-methylbutanal were also observed after roasting. The spectral peak was the most obvious in the meat of large yellow croaker roasted with 45% light waves and 55% microwaves.

Aldehydes are important contributors to fish flavor and are mainly derived from the oxidation and degradation of lipids in fish. They are one of the most typical odors in aquatic products. This was in agreement with the evaluation of steamed Patin catfish by Pratama et al. [53]. The threshold for aldehydes to produce fruity, grassy, and other odor characteristics is low, and this contributes to the many characteristic flavors of fish. Fatty octanal was detected in the fish prepared using the five roasting methods, which was likely due to lipid oxidation. 2-Methylbutyraldehyde is produced by the Maillard reaction during heating, and it produces the characteristic smell of roasted fish. The production of isovaleraldehyde also has an important influence on the flavor of food. The roasting temperature and time are key factors affecting the oxidation of polyunsaturated fatty acids in meat [54].

#### 3.4.4. Alcohols in Large Yellow Croaker Prepared Using Different Roasting Methods

Eight alcohols were detected in the large yellow croaker meat. 3-Pentanol, eucalyptol, 3-methyl-2-butanol, and 2-methyl-1-propanol appeared after the meat was roasted. Among them, eucalyptol only appeared in fish roasted with 45% light waves and 55% microwaves. Alcohols play a vital role in the overall flavor of fish. This was mainly due to lipid oxidation and the Strecker degradation reaction. High-temperature processing induces the degradation of linoleic acid hydroperoxide in fish. The alcohols usually have a pleasant fruity and aromatic flavor, and the eucalyptol has a light mint fragrance.

#### 3.4.5. Ketones in Yellow Croaker Prepared Using Different Roasting Methods

Twelve ketones were detected in the large yellow croaker meat. Diketones are the primary products of the Maillard reaction and produce meat flavors. 2,3-Butanedione was found in the fish after roasting. 2,3-Pentanedione was only found in fish roasted using 45% light waves and 55% microwaves, and it is the key aroma substance of large yellow croaker roasted fish. Ketones are produced by thermal oxidative degradation of unsaturated fatty acids or amino acids. Unsaturated fatty acids or amino acids mainly produce eucalyptus, fruit, and fatty odors. The production of ketones can reduce the fishy odor [55,56]. The ketone content of the roasted fish was higher than that of the raw fish. Therefore, the fishy odor of the fish can be reduced by high-temperature roasting, and the odor value of the sensory evaluation score after roasting was higher than that of the raw fish.

#### 3.4.6. Esters in Large Yellow Croaker Prepared Using Different Roasting Methods

Ten esters were detected in the meat of large yellow croakers. Esters were the most abundant in the 45% light wave and 55% microwave roasting method, followed by the 70% light wave and 30% microwave method. Methyl butyrate and methyl hexanoate appeared after the fish was roasted. Ethyl butyrate and ethyl (E)-2-methyl-2-pentenoate were only detected in large yellow croaker meat baked with 45% light waves and 55% microwaves. 

Esters are produced by lipid metabolism or the esterification of alcohols and carboxylic acids. Ester compounds usually have a pleasant fruity sweetness. Ester compounds appeared only after high-temperature roasting, indicating that large yellow croaker fish could give a certain aroma after roasting. This was in agreement with the evaluation of grilled eel volatile fingerprinting by Huang et al. using e-Nose, GC-O, GC-MS, and GC x GC-QTOF combined with purge and trap and solvent-assisted flavors [57].

#### 3.4.7. Olefins and Other Substances in Large Yellow Croaker Fish Roasted by Different Roasting Methods

In this study, the production of several substances was related to the Maillard reaction. Acetic acid is a landmark product of the 2,3-enolization pathway of the Maillard reaction. Two types of furan compounds were also detected, which were produced by the thermal degradation of carbohydrates, the Maillard reaction, and lipid oxidation [58]. These two compounds were 2-ethylfuran and 2-pentylfuran, respectively, but their contents were low. 2-pentylfuran was highly perceptible, with bean, fruit, green, and vegetable aromas.

**Figure 4:**
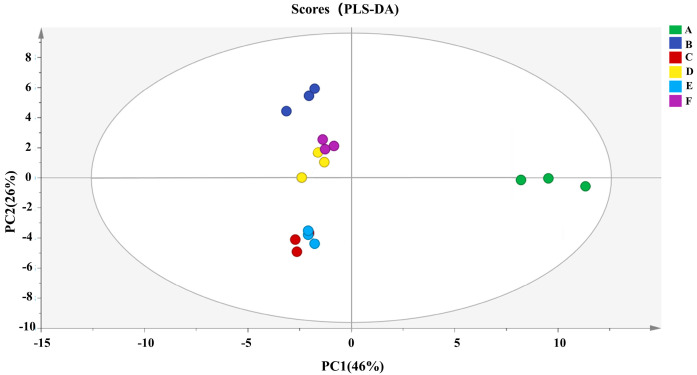
Partial least squares discriminant analysis (PLS−DA) score plot of key volatile flavor compounds in large yellow croaker prepared using different roasting methods. A−F represent different roasting methods. A−control, B−45% light wave and 55% microwave roasting, C−70% light wave and 30% microwave roasting, D−100% light wave roasting, E—far-infrared roasting, and F−oven roasting.

To more effectively distinguish the differences between the samples and determine the important variables that led to these differences, partial least squares discriminant analysis (PLS-DA) was used to further study the volatile flavor substances detected by GC-IMS [59]. The fitting parameters of the PLS-DA model (R2X = 0.456 and R2Y = 0.266) showed a strong explanatory power. As shown in Figure 4, of the five principal components of the PLS-DA model, PC1 accounted for 46% of the variable information, and PC2 accounted for 26%, which may help us to understand the correlation and discrimination of the variables. In this model, raw fish and fish samples roasted using different methods were distributed on the right and left sides of PC1, indicating that there were significant differences between the roasted fish and raw fish groups. Among them, the corresponding values of 100% light wave roasting (group D) and oven roasting (group F) were similar, whereas the corresponding values of 70% light wave and 30% microwave roasting (group C) and far-infrared roasting (group E) were similar.

### 3.5. Effects of Different Roasting Methods on Sensory Evaluation of Large Yellow Croaker Fish

**Figure 5:**
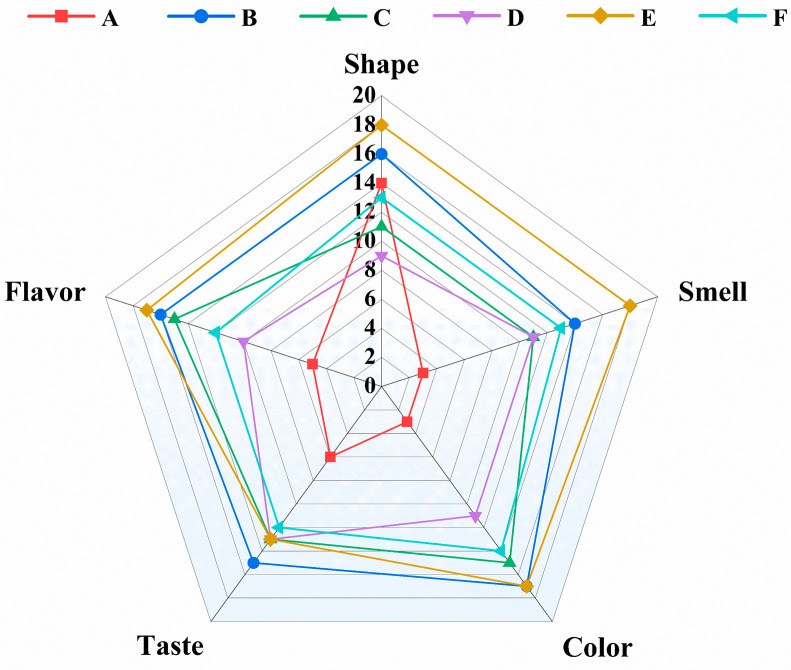
Radar chart of sensory evaluation scores for different methods of roasting large yellow croaker fish. A–F represent different roasting methods. A—Control, B—45% light wave and 55% microwave roasting, C—70% light wave and 30% microwave roasting, D—100% light wave roasting, E—far-infrared roasting, and F—oven roasting.

Sensory quality is an indicator that can be used to directly characterize the flavor of fried fish using different roasting methods. After roasting, fish meat shows significant improvements in appearance, smell, and taste [60]. The sensory evaluation of fish fried using the five different methods was carried out based on five aspects: shape, smell, color, taste, and acceptance. The results are shown in Figure 5.

Compared with raw large yellow croaker fish, among the five different roasted large yellow croaker fish samples, the overall sensory score of the far-infrared-roasted large yellow croaker fish was the highest, at 83.1 points. The lowest score was 53.1 for 100% light wave-baked fish. As shown in Figure 5, the color, taste, and smell of the large yellow croaker meat significantly improved after roasting, and acceptance also improved. Group E (far-infrared-baked large yellow croaker meat) had the highest odor, color, and shape scores. The three types of light wave–microwave combination-baked fish showed large differences in color and smell, but the taste attribute scores were similar between these groups. The taste scores were in the order of light wave and microwave roasting > far-infrared roasting > oven roasting; the smell scores were in the order of far-infrared roasting > oven roasting > light wave and microwave roasting; and the overall acceptance scores were in the order of far-infrared roasting > light wave and microwave roasting > oven roasting.

In general, the sensory evaluation showed that the surface color of far-infrared-baked large yellow croaker fish was golden, the shape was complete, and the muscle was tender, delicious, and highly acceptable. Fish roasted with 45% light wave and 55% microwave had a golden color and the best taste. The morphology of large yellow croaker baked using 100% light wave was soft, the color was dim, and the smell was slightly fishy, but the meat was full of juice and was dense. This is also consistent with the above research results on texture, color difference, moisture, and flavor, and provides a reference for the optimization of the big yellow croaker baking process.

## 4. Conclusions

In summary, different roasting methods have significant effects on the texture, color difference, moisture content, and flavor characteristics of large yellow croaker fish. Compared with raw fish, different roasting methods gave the fish a golden yellow color, reduced the hardness of the fish, and gave the fish elasticity. The far-infrared-roasted fish was golden in color. The 45% light wave- and 55% microwave-roasted fish had good meat quality, the best flavor, a reduced content of immobilized water and free water, and produced the most volatile flavor substances. Among them, ethyl acetate, ethyl butyrate, and hexyl acetate could give the fish a fruity and sweet taste. Octanal with a fat flavor was detected in the roasted fish of large yellow croaker after all five roasting methods, which was probably due to lipid oxidation. 2-Methylbutyraldehyde is produced by the Maillard reaction during heating, which can give the fish the smell of roasting. The results showed that the 45% light wave and 55% microwave roasting method had a more positive effect on the texture improvement, water stability, and aromatic active component formation of large yellow croaker meat and had more value in the improvement of the quality of large yellow croaker roasted fish, but it also needed to be optimized in terms of the roasting time.

In addition, according to the results obtained, and compared with other baking methods, the microwave–light wave baking method has a shorter time and lower energy consumption and has a good prospects for commercial production in the process of grilled fish processing. In future research, we will introduce the effects of the same microwave and light wave combined heating technology on the non-volatile flavor substances, healthy lipid index, and nutritional characteristics of large yellow croaker grilled fish meat and provide a further reference for the commercial production of grilled fish.

## Figures and Tables

**Table 1:** Sensory evaluation criteria of large yellow croaker grilled fish for different roasting methods.

Score	Shape	Smell	Color	Taste	Acceptance
16–20	The morphology is particularly complete, and the hand-tear section has clear muscle fibers, full of elasticity.	The grilled fish flavor is very obvious, with no fishy smell.	The color is brighter, and the surface is shinier, with a very well-baked color.	The meat is moderate in hardness, good in chewiness, and good in taste.	The overall feeling is very good, with no unpleasant sensory experiences.
11–15	The morphology is complete, and the hand-tear section has slightly clear muscle fibers and is elastic.	The roast fish flavor is more obvious, with a slight fishy taste.	The color is brighter, and the surface is shinier, with a slightly baked color.	The meat is slightly hard or soft, the chewiness is good, and the taste is acceptable.	The overall feeling is better; less unpleasant sensory experience.
6–10	The morphology is slightly complete, and the hand-tear section has no clear muscle fibers, with poor elasticity.	The roast fish flavor is not obvious, and the fishy smell heavier.	The fish has a dark color, and the surface has no obvious luster and is slightly pasty.	The meat has poor chewing ability, with a very rough texture.	The overall feeling is acceptable, and the unpleasant sensory experience is more obvious.
0–5	The morphology is incomplete and inelastic.	No grilled fish flavor, and the fishy smell is heavy.	The surface has no color and luster, and looks like burnt char.	The meat is hard or soft, loses chewiness, and has a poor taste.	The overall feeling is very poor, and the sensory discomfort is very obvious.

**Table 2:** The color difference values of large yellow croaker grilled fish for different roasting methods.

Roasting Method	L*	a*	b*
A	49.44 ± 3.65 **^a^**	0.50 ± 1.50 **^b^**	3.86 ± 2.37 **^a^**
B	40.39 ± 1.48 **^b^**	1.21 ± 0.10 **^b^**	6.63 ± 1.48 **^a^**
C	28.34 ± 3.39 **^c^**	2.34 ± 1.11 **^ab^**	6.46 ± 3.86 **^a^**
D	29.13 ± 5.35 **^c^**	5.90 ± 4.37 **^a^**	6.71 ± 0.55 **^a^**
E	41.18 ± 6.41 **^ab^**	1.55 ± 0.48 **^b^**	6.49 ± 3.14 **^a^**
F	41.36 ± 4.60 **^ab^**	2.57 ± 0.42 **^ab^**	9.51 ± 2.97 **^a^**

Note: The same lowercase letter indicates that there is no significant difference between different roasting methods (*p* > 0.05), whereas different lowercase letters indicate significant differences between different roasting methods (*p* < 0.05).”a”, “b”, “c” represents a significant difference between the groups. “a” and “ab” contain the same letter “a”, indicating no significant difference between the two groups. “b” in “ab” indicates no significant difference between this group and other groups containing the letter “b”. A–F represent different roasting methods. A—control, B—45% light wave and 55% microwave roasting, C—70% light wave and 30% microwave roasting, D—100% light wave roasting, E—far-infrared roasting, and F—oven roasting.

**Table 3:** The texture characteristics of large yellow croaker roasted using different roasting methods.

Type	Springiness/mm	Hardness/N	Chewiness/mJ	Gumminess/N
A	1.06 ± 0.01 **^a^**	6.34 ± 1.38 **^a^**	2.22 ± 1.24 **^a^**	2.22 ± 0.97 **^a^**
B	0.93 ± 0.80 **^a^**	2.28 ± 0.16 **^b^**	0.87 ± 0.21 **^b^**	1.08 ± 0.03 **^b^**
C	0.89 ± 0.15 **^a^**	2.47 ± 0.25 **^b^**	1.03 ± 0.21 **^ab^**	1.18 ± 0.32 **^b^**
D	0.80 ± 0.08 **^a^**	3.16 ± 0.07 **^b^**	1.25 ± 0.58 **^ab^**	1.55 ± 0.39 **^ab^**
E	0.75 ± 0.28 **^a^**	2.33 ± 0.55 **^b^**	0.97 ± 0.60 **^b^**	1.20 ± 0.33 **^b^**
F	0.78 ± 0.13 **^a^**	2.64 ± 0.22 **^b^**	1.21 ± 0.42 **^ab^**	1.33 ± 0.51 **^ab^**

Note: The same lowercase letter indicates that there is no significant difference between different roasting methods (*p* > 0.05), whereas different lowercase letters indicate significant differences between different roasting methods (*p* < 0.05). “a”, “b” represents a significant difference between the groups. “a” and “ab” contain the same letter “a”, indicating no significant difference between the two groups. “b” in “ab” indicates no significant difference between this group and other groups containing the letter “b”. A–F represent different roasting methods. A—control, B—45% light wave and 55% microwave roasting, C—70% light wave and 30% microwave roasting, D—100% light wave roasting, E—far-infrared roasting, and F—oven roasting.

**Table 4:** T_2_ relaxation time of large yellow croaker prepared with different roasting methods.

Roasting Method	T_21_ (ms)	T_22_ (ms)	T_23_ (ms)
A	0.21 ± 0.02 **^ab^**	33.70 ± 0.00 **^a^**	821.43 ± 0.00 **^a^**
B	0.25 ± 0.02 **^a^**	25.53 ± 0.00 **^c^**	296.89 ± 12.03 **^e^**
C	0.25 ± 0.02 **^a^**	29.33 ± 0.00 **^b^**	357.08 ± 0.00 **^d^**
D	0.17 ± 0.01 **^b^**	25.54 ± 0.00 **^c^**	506.08 ± 35.11 **^b^**
E	0.18 ± 0.04 **^b^**	21.52 ± 0.06 **^d^**	332.95 ± 1.31 **^d^**
F	0.18 ± 0.01 **^b^**	29.33 ± 0.00 **^b^**	471.38 ± 0.00 **^c^**

Note: The same lowercase letter indicates that there is no significant difference between different roasting methods (*p* > 0.05), whereas different lowercase letters indicate significant differences between different roasting methods (*p* < 0.05). “a”, “b”, “c”, “d”, “e” represents a significant difference between the groups. “a” and “ab” contain the same letter “a”, indicating no significant difference between the two groups.”b” in “ab” indicates no significant difference between this group and other groups containing the letter “b”. A–F represent different roasting methods. A—control, B—45% light wave and 55% microwave roasting, C—70% light wave and 30% microwave roasting, D—100% light wave roasting, E—far-infrared roasting, and F—oven roasting.

**Table 5:** Water peak surface area values of large yellow croaker grilled using different roasting methods.

Roasting Method	A_21_	A_22_	A_23_
A	1260.83 ± 46.63 ^b^	18,270.86 ± 42.78 ^a^	1090.93 ± 10.09 ^d^
B	745.95 ± 82.12 ^d^	13,790.28 ± 29.42 ^d^	714.28 ± 16.65 ^e^
C	838.22 ± 20.48 ^c^	11,390.85 ± 16.97 ^f^	502.23 ± 10.34 ^f^
D	1732.11 ± 24.09 ^a^	11,832.85 ± 37.82 ^e^	6033.21 ± 5.73 ^a^
E	1231.07 ± 36.54 ^b^	14,189.50 ± 23.07 ^c^	3753.89 ± 37.78 ^c^
F	1685.29 ± 20.53 ^a^	16,105.62 ± 33.69 ^b^	5480.20 ± 1.36 ^b^

Note: The same lowercase letter indicates that there is no significant difference between different roasting methods (*p* > 0.05), whereas different lowercase letters indicate significant differences between different roasting methods (*p* < 0.05). “a”, “b”, “c”, “d”, “e”, “f” represents a significant difference between the groups. A–F represent different roasting methods. A—control, B—45% light wave and 55% microwave roasting, C—70% light wave and 30% microwave roasting, D—100% light wave roasting, E—far-infrared roasting, and F—oven roasting.

**Table 6:** Qualitative information for large yellow croaker samples prepared using different roasting methods.

Type	Count	Compound	CAS#	Formula	MW	RI	Odor Description
**Aldehydes**	1	Pentanal	110-62-3	C_5_H_10_O	86.1	958.8	Almond, green, malty
	2	(E)-2-Hexenal	6728-26-3	C_6_H_10_O	98.1	1191.8	Green, fatty, leafy
	3	3-Methylbutanal	590-86-3	C_5_H_10_O	86.1	914.1	Ethereal, chocolate, peach, fatty
	4	Octanal	124-13-0	C_8_H_16_O	128.2	1283.3	Grease, citrus, soapy
	5	Butanal	123-72-8	C_4_H_8_O	72.1	866.6	Banana, pungent
	6	2-Methylbutanal	96-17-3	C_5_H_10_O	86.1	909.6	Cocoa, fermented
Ketones	7	2-Propanone	67-64-1	C_3_H_6_O	58.1	810.2	Solvent, ethereal
	8	5-Methyl-2-heptanone	18217-12-4	C_8_H_16_O	128.2	1256	
	9	3-Penten-2-one	625-33-2	C_5_H_8_O	84.1	1132.1	Fruity, phenolic, fishy
	10	Pentan-2-one (M)	107-87-9	C_5_H_10_O	86.1	1015.8	Oily, fruity, fusel
	11	Pentan-2-one (D)	107-87-9	C_5_H_10_O	86.1	976.5	
	12	2-Butanone	78-93-3	C_4_H_8_O	72.1	891	Butterscotch, ether
	13	Acetone	67-64-1	C_3_H_6_O	58.1	801.5	Solvent, apple, pear
	14	2-Heptanone (M)	110-43-0	C_7_H_14_O	114.2	1177.3	Cheese, fruity
	15	2,3-Pentanedione	600-14-6	C_5_H_8_O_2_	100.1	1061.9	Pungent, sweet, nutty
	16	2,3-Butanedione	431-03-8	C_4_H_6_O_2_	86.1	1023.9	Sweet, creamy, buttery
	17	2-Butanone-3-hydroxy (M)	513-86-0	C_4_H_8_O_2_	88.1	1288.1	Buttery
	18	2-Butanone-3-hydroxy (D)	513-86-0	C_4_H_8_O_2_	88.1	1284.4	
	19	1-Hydroxy-2-propanone	116-09-6	C_3_H_6_O_2_	74.1	1297.8	Pungent, ethereal
	20	2-Pentanone	107-87-9	C_5_H_10_O	86.1	1021.2	Banana, woody, wine
	21	2-Heptanone (D)	110-43-0	C_7_H_14_O	114.2	1189.1	Green, banana
Alcohols	22	1-Hexanol (M)	111-27-3	C_6_H_14_O	102.2	1361.5	Fruity, alcoholic
	23	1-Hexanol (D)	111-27-3	C_6_H_14_O	102.2	1357.7	
**Oi**	24	Hydroxyacetone	116-09-6	C_3_H_6_O_2_	74.1	1284.9	Caramel-like, ethereal
	25	2-Methyl-1-propanol (M)	78-83-1	C_4_H_10_O_2_	74.1	1100.8	Slightly suffocating, non-residual alcoholic
	26	2-Methyl-1-propanol (D)	78-83-1	C_4_H_10_O_2_	74.1	1094.8	
	27	1-Pentanol (M)	71-41-0	C_5_H_12_O	88.1	1259.2	Pungent, yeasty, winey
	28	1-Pentanol (D)	71-41-0	C_5_H_12_O	88.1	1237.5	
	29	3-Pentanol	584-02-1	C_5_H_12_O	88.1	1112.5	Sweet, herbal
	30	1,8-Cineole	470-82-6	C_10_H_18_O	154.3	1189.1	Eucalyptus, medicinal
	31	3-Methyl-2-butanol (M)	598-75-4	C_5_H_12_O	88.1	1080.3	Fruity
	32	3-Methyl-2-butanol (D)	598-75-4	C_5_H_10_O	88.1	1147.4	
	33	2-Methyl-1-propanol	78-83-1	C_4_H_10_O	74.1	1111.6	Ethereal, wine
**Esters**	34	Ethyl propanoate	105-37-3	C_5_H_10_O_2_	102.1	942.3	Bubblegum, grape
	35	Isopropyl acetate	108-21-4	C_5_H_10_O_2_	102.1	897.4	Ethereal, chemical
	36	Methyl caproate	106-70-7	C_7_H_14_O_2_	130.2	1189.8	Fruity, fatty, apricot
	37	Methyl hexanoate	106-70-7	C_7_H_14_O_2_	130.2	1178.8	Pineapple, tropical
	38	Ethyl butanoate	105-54-4	C_6_H_12_O_2_	116.2	1045	Tutti, fresh, sweet
	39	Ethyl (E)-2-methyl-2-pentenoate	1617-40-9	C_8_H_14_O_2_	142.2	1142.4	
	40	Methyl butanoate	623-42-7	C_5_H_10_O_2_	102.1	988.1	Apple, pineapple
	41	Iso-Propyl acetate	108-21-4	C_5_H_10_O_2_	102.1	907.3	
	42	Hexyl acetate	142-92-7	C_8_H_16_O_2_	144.2	1278.4	Fruity, sweet
	43	Ethyl Propanoate	105-37-3	C_5_H_10_O_2_	102.1	958.8	Sweet, rummy, juicy
	44	Ethyl Acetate	141-78-6	C_4_H_8_O_2_	88.1	848.6	Balsamic, fruit, grape, pineapple
Enolefins	45	Alpha-pinene (M)	80-56-8	C_10_H_16_	136.2	1040.9	Fresh, pine, woody
	46	Alpha-pinene (D)	80-56-8	C_10_H_16_	136.2	998.9	
	47	1-Octene	111-66-0	C_8_H_16_	112.2	827.1	Gasoline
	48	Styrene	100-42-5	C_8_H_8_	104.2	1232.4	Balsamic, gasoline
	49	1-Heptene	592-76-7	C_7_H_14_	98.2	751.3	Sweet
	50	Limonene (M)	138-86-3	C_10_H_16_	136.2	1194.9	Terpenic
	51	Limonene (D)	138-86-3	C_10_H_16_	136.2	1232.7	
Others	52	4-Ethyl-M-xylene	874-41-9	C_10_H_14_	134.2	1346.4	
	53	P-Cymene	99-87-6	C_10_H_14_	134.2	1261.6	Fresh, citrus, woody
	54	Acetic acid (M)	64-19-7	C_2_H_4_O_2_	60.1	1471.9	Sharp, pungent, sour
	55	Acetic acid (D)	64-19-7	C_2_H_4_O_2_	60.1	1471.9	
	56	2-Pentylfuran	3777-69-3	C_9_H_14_O	138.2	1222.8	Floral, fruit, green bean
	57	2-Ethyl furan	3208-16-0	C_6_H_8_O	96.1	962.8	Sweet, burnt, malty

Note: MW: molecular mass. RI: retention index. M: monomer. D: dimer. The odor descriptions of the volatile flavor compounds were referenced using the Perflavory Information System.

## Data Availability

Data are contained within the article.
